# The Effect of Pressure and Solvent on the Supercritical Fluid Chromatography Separation of Tocol Analogs in Palm Oil

**DOI:** 10.3390/molecules22091424

**Published:** 2017-08-29

**Authors:** Mei Han Ng, Ahmad Kushairi

**Affiliations:** Engineering and Processing Research Division, Malaysian Palm Oil Board, 6, Persiaran Institusi, Bandar Baru Bangi, Kajang 43000, Selangor, Malaysia; kushairi@mpob.gov.my

**Keywords:** palm, supercritical fluid chromatography, tocol analogs, tocotrienol

## Abstract

There are six tocol analogs present in palm oil, namely α-tocopherol (α-T), α-tocomonoenol (α-T_1_), α-tocotrienol (α-T_3_), γ-tocotrienol (γ-T_3_), β-tocotrioenol (β-T_3_) and δ-tocotrienol (δ-T_3_). These analogs were difficult to separate chromatographically due to their similar structures, physical and chemical properties. This paper reports on the effect of pressure and injection solvent on the separation of the tocol analogs in palm oil. Supercritical CO_2_ modified with ethanol was used as the mobile phase. Both total elution time and resolution of the tocol analogs decreased with increased pressure. Ethanol as an injection solvent resulted in peak broadening of the analogs within the entire pressure range studied. Solvents with an eluent strength of 3.4 or less were more suitable for use as injecting solvents.

## 1. Introduction

**Figure 1:**
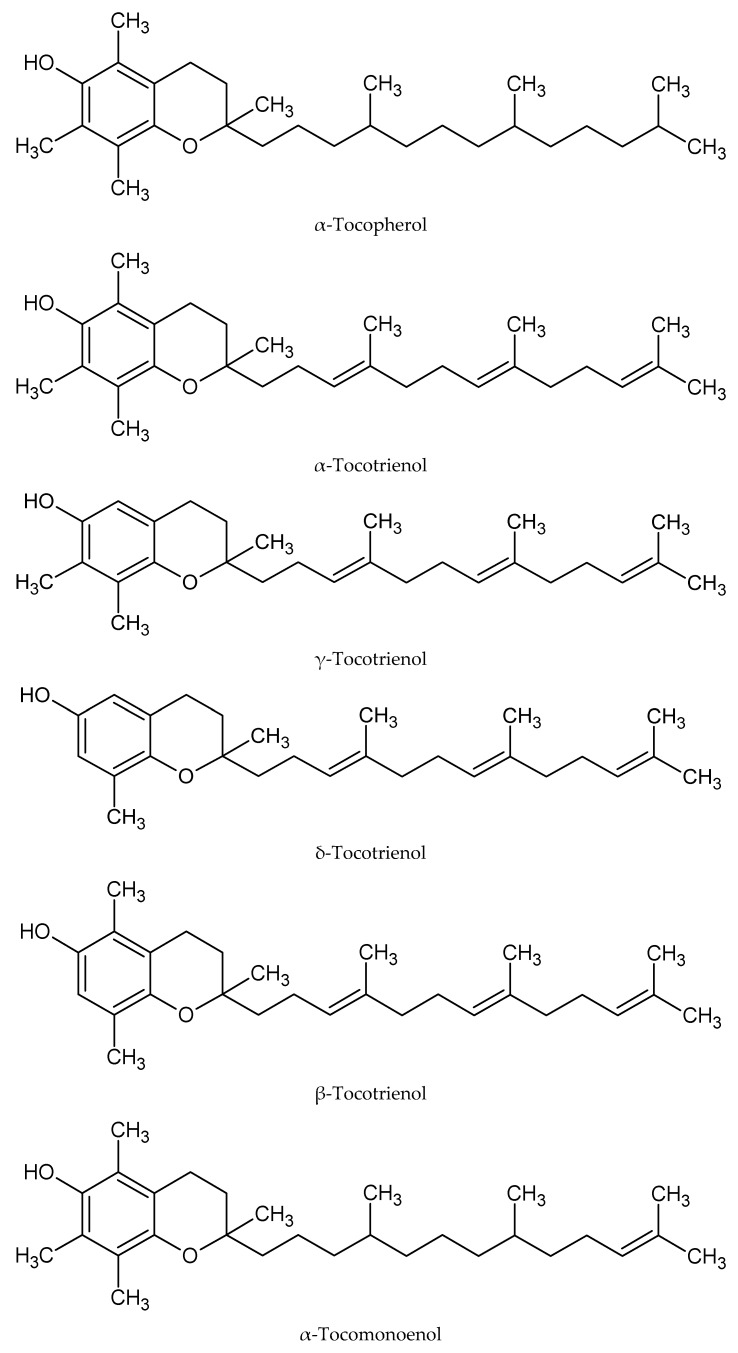
Molecular structure of tocol analogs found in palm oil.

There are six types of tocols in palm oil, namely α-tocopherol (α-T), α-tocomonoenol (α-T_1_), α-tocotrienol (α-T_3_), γ-tocotrienol (γ-T_3_), β-tocotrioenol (β-T_3_) and δ-tocotrienol (δ-T_3_) [1,2,3,4,5,6,7,8]. All these tocols are structural analogs. They have a common chromanol structure with different side chains. Tocopherol has a saturated side chain, tocomonoenol is mono-unsaturated, while tocotrienol has three unsaturated bonds at its side chain. The alpha, beta, gamma or delta forms, on the other hand, are determined by the type and position of the methyl group substituent in the main chromanol structure (Figure 1).

The tocol analogs exhibit similar physical, chemical, biochemical and pharmacological properties [1,8,9]. Most often, these tocol analogs cannot be distinguished due to their high physical and chemical property similarity.

Supercritical fluid chromatography (SFC) is a highly efficient technique for the separation of otherwise chromatographically challenged compounds. SFC is a powerful separation technique, incorporating the high diffusivity, low viscosity and high solvating power of gas chromatography (GC) and high performance liquid chromatography (HPLC) [4,10,11,12]. The unique properties of a supercritical fluid as a mobile phase overcome the difficulties of solute thermal instability and volatility encountered in GC and also shorten the relatively long analyses times of HPLC separations [10,12,13,14]. SFC is particularly known for its superior separation of chiral compounds in terms of speed and resolution [11,15,16,17].

SFC has been used in the past for analyses of palm oil components [4,6,18,19,20,21]. SFC of carotenes, vitamin E and sterols has also been reported in the past using model mixtures. However, it is noteworthy that SFC separations are very different in a real world sample matrix compared to model matrixes where interferences from other compounds were eliminated. Choo et al. reported a straightforward SFC protocol, in terms of elution, squalene, carotenes, vitamin E and sterols, from real palm oil samples in a single run [4].

SFC for palm-based solutes is often carried out with supercritical CO_2_ as the mobile phase. CO_2_ is a non-polar solvent, and consequently needs an organic modifier to facilitate the elution of more polar solutes. Peaks eluted from lower to higher polarity in a normal stationary phase under such mobile phase. Most SFC applications in the early days involved the separation of non-polar solutes and homologue series. SFC is thus thought to be a suitable chromatographic tool for the separation of the tocol analogs in palm oil. The tocol analogs are readily dissolved in non-polar to moderate polarity organic solvents. Ideally chromatographic samples should be dissolved in the mobile phase to avoid addition of a third solvent. However, the tocols do not dissolve in ethanol, which is the modifier in the SFC separation. Thus, it is interesting to know the effect of different injecting solvents in SFC. This is because each solvent has its own eluent strength, which is a measure of the solvent adsorption energy, with the value for pentane defined as 0 on bare silica. The more polar the solvent, the greater is its eluent strength and the more rapidly will solutes be eluted from the column. The effect of pressure on the SFC separation of the tocol analogs was also studied and reported in this study.

## 2. Results

**Figure 2:**
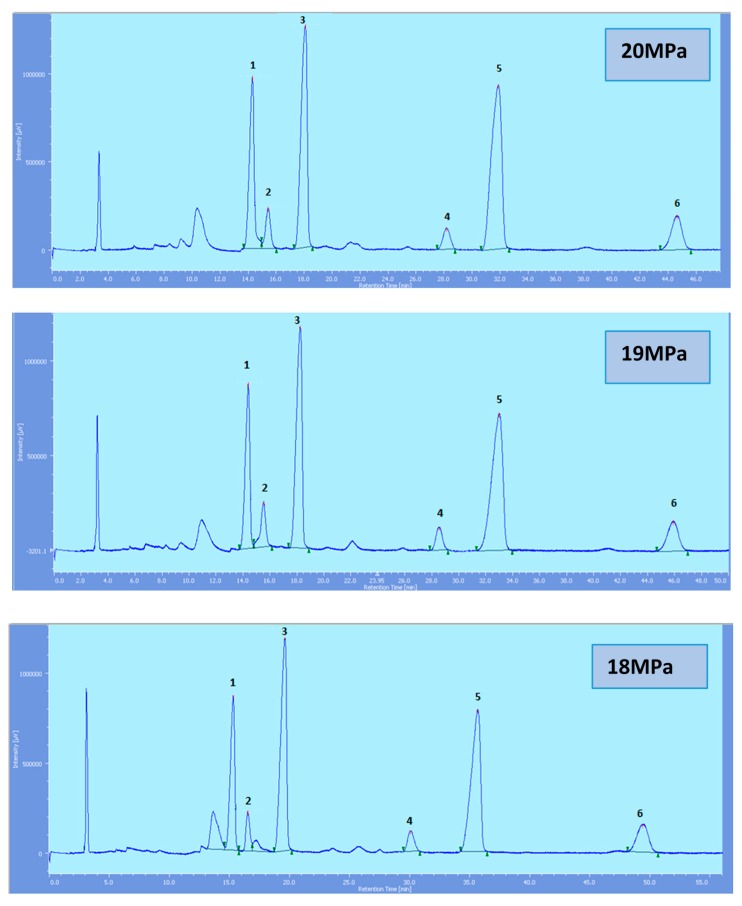

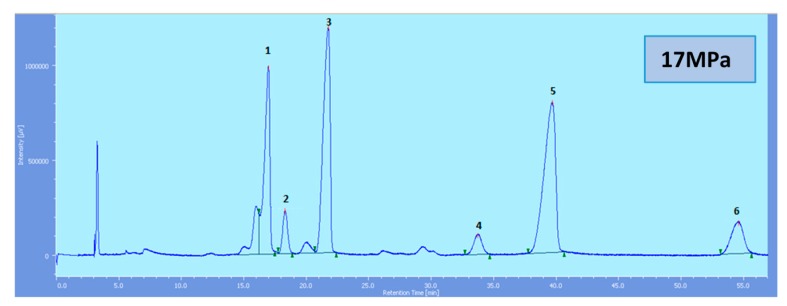
SFC of tocols dissolved in *n*-heptane. 1: α-T, 2: α-T_1_, 3: α-T_3_, 4: β-T_3_, 5: γ-T_3_, 6: δ-T_3_.

**Figure 3:**
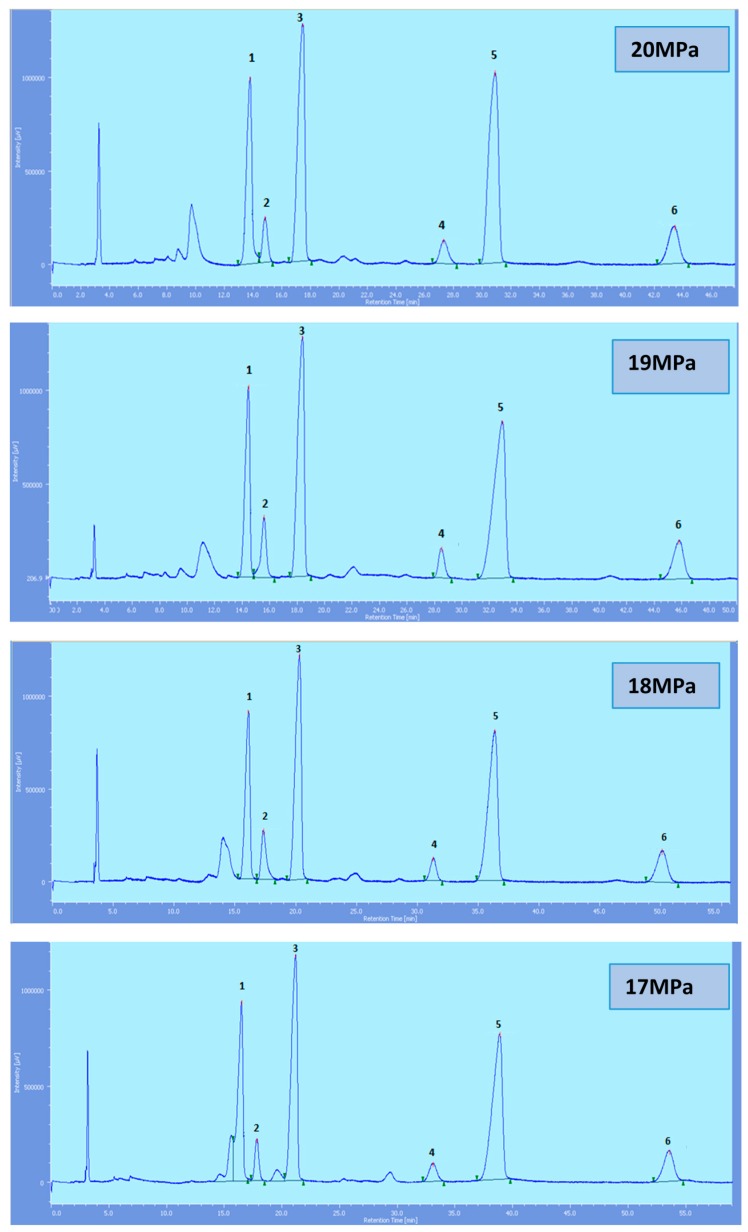
SFC of tocols dissolved in *n*-hexane. 1: α-T, 2: α-T_1_, 3: α-T_3_, 4: β-T_3_, 5: γ-T_3_, 6: δ-T_3_.

**Figure 4:**
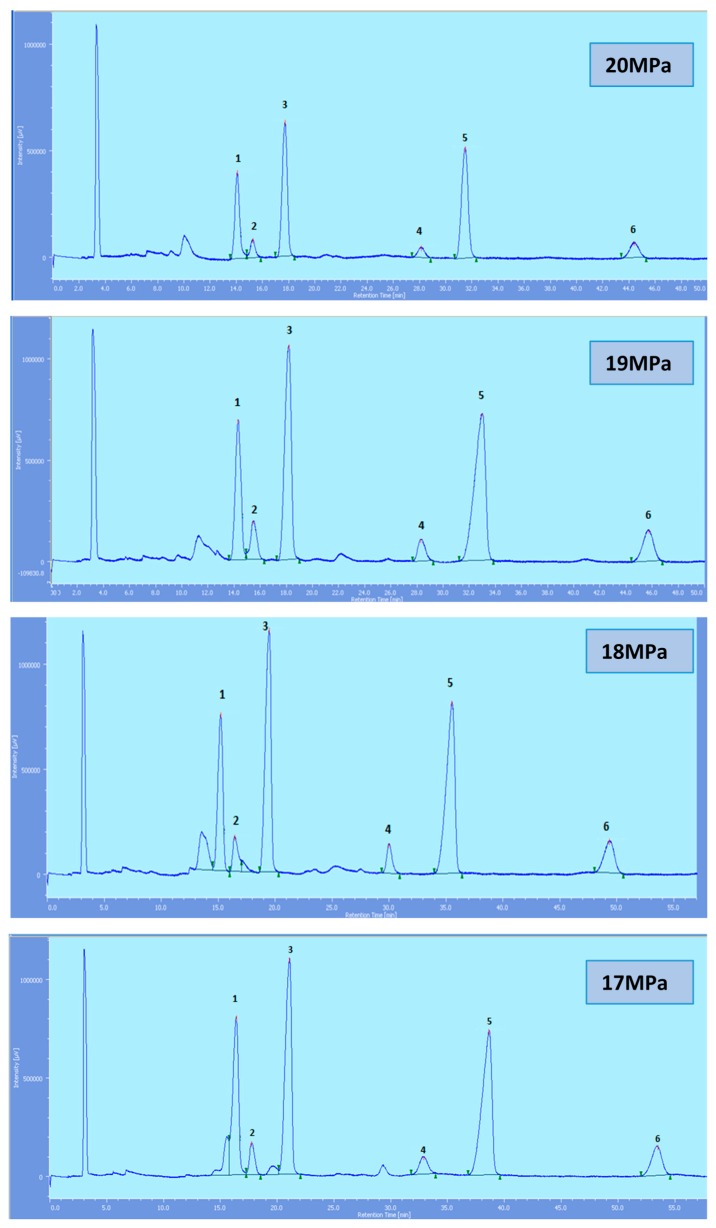
SFC of tocols dissolved in DCM. 1: α-T, 2: α-T_1_, 3: α-T_3_, 4: β-T_3_, 5: γ-T_3_, 6: δ-T_3_.

**Figure 5:**
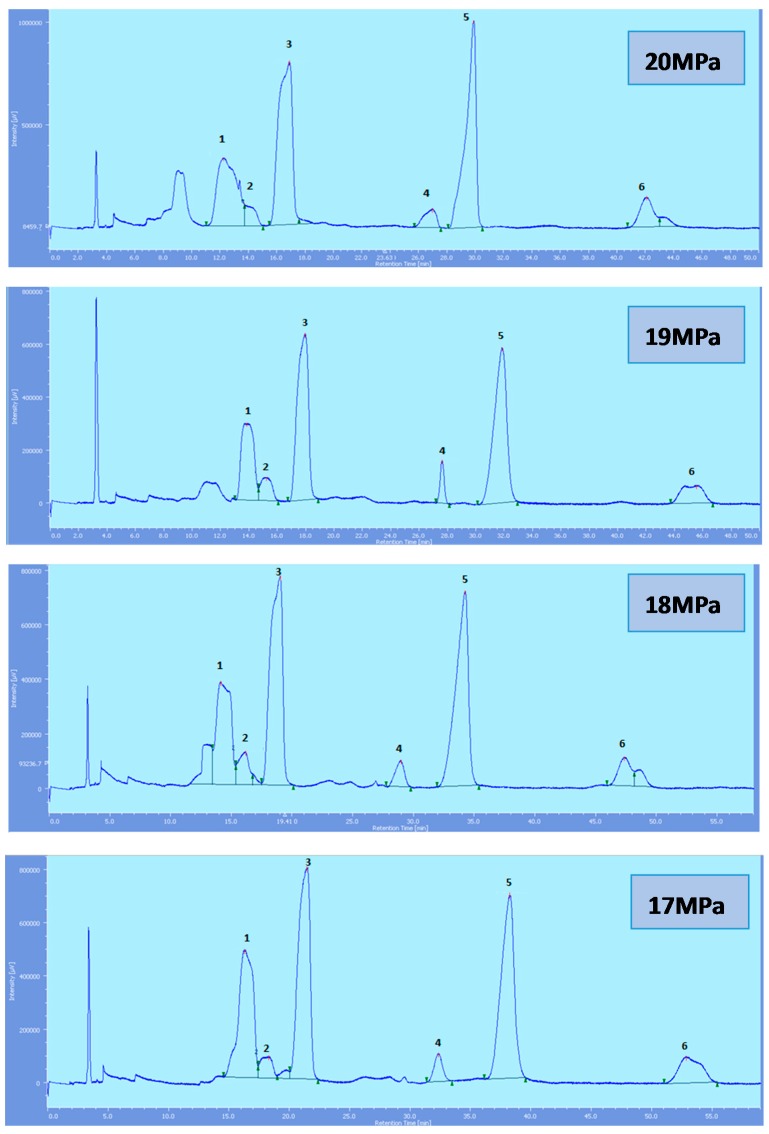
SFC of tocols dissolved in ethanol. 1: α-T, 2: α-T_1_, 3: α-T_3_, 4: β-T_3_, 5: γ-T_3_, 6: δ-T_3_.

**Figure 6:**
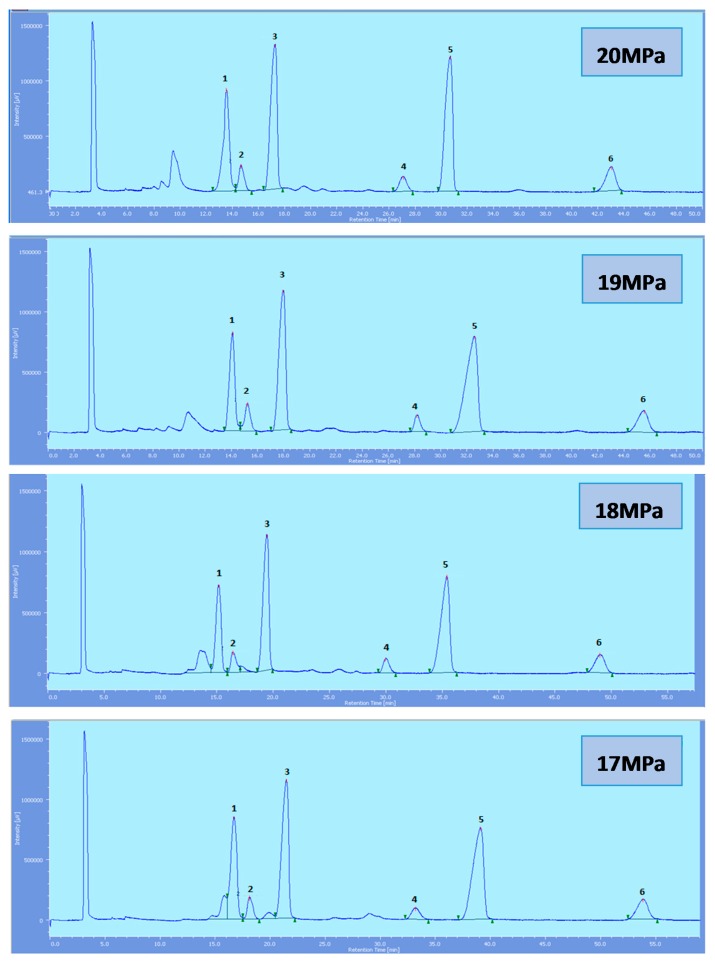
SFC of tocols dissolved in chloroform. 1: α-T, 2: α-T_1_, 3: α-T_3_, 4: β-T_3_, 5: γ-T_3_, 6: δ-T_3_.

Supercritical CO_2_ modified with ethanol is able to elute all the tocol analogs present in palm oil. The elution of the tocol analogs on the silica column follows the order of α-T, α-T_1_, α-T_3_, β-T_3_, γ-T_3_ and δ-T_3_ (Figure 2, Figure 3, Figure 4, Figure 5 and Figure 6).

**Figure 7:**
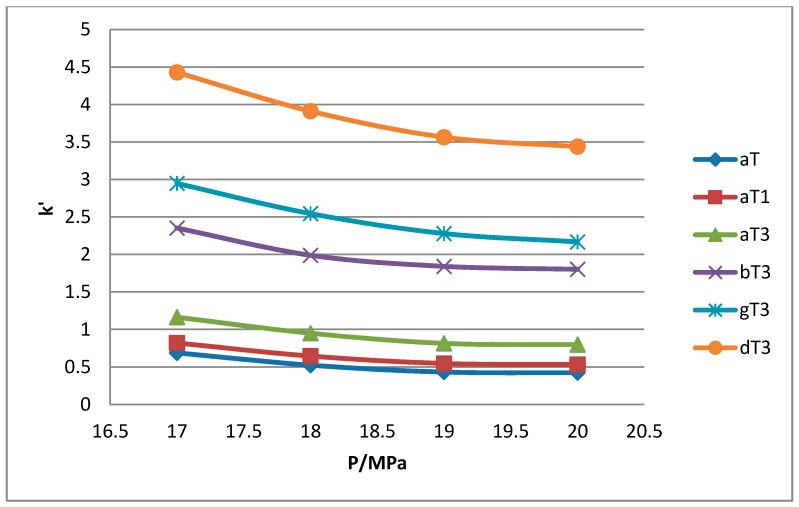
Effect of pressure on capacity factor for tocols dissolved in *n*-heptane.

**Figure 8:**
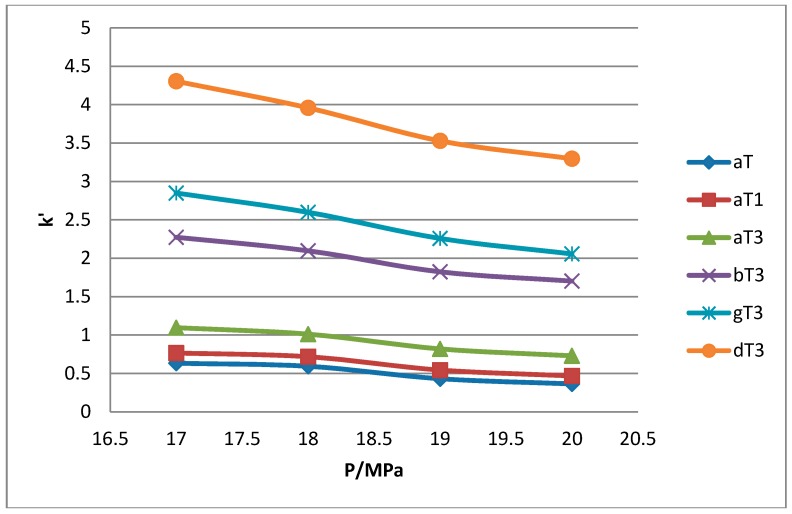
Effect of pressure on capacity factor for tocols dissolved in *n*-hexane.

**Figure 9:**
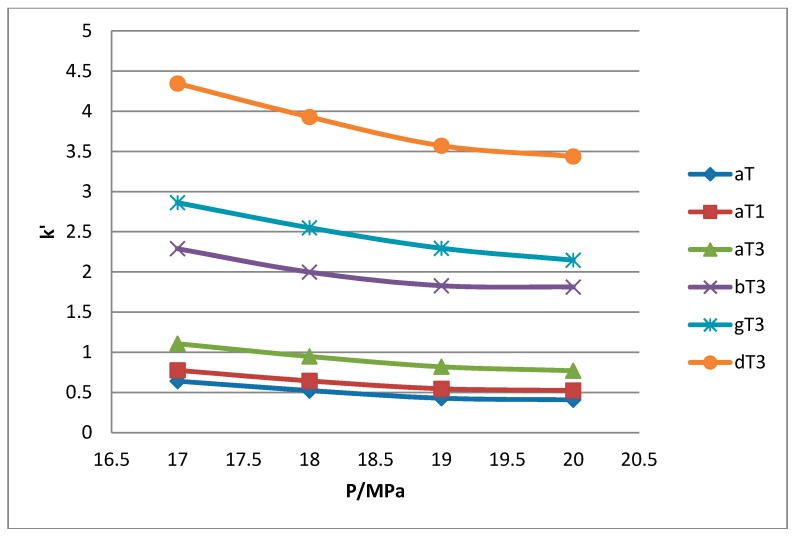
Effect of pressure on capacity factor for tocols dissolved in dichloromethane.

**Figure 10:**
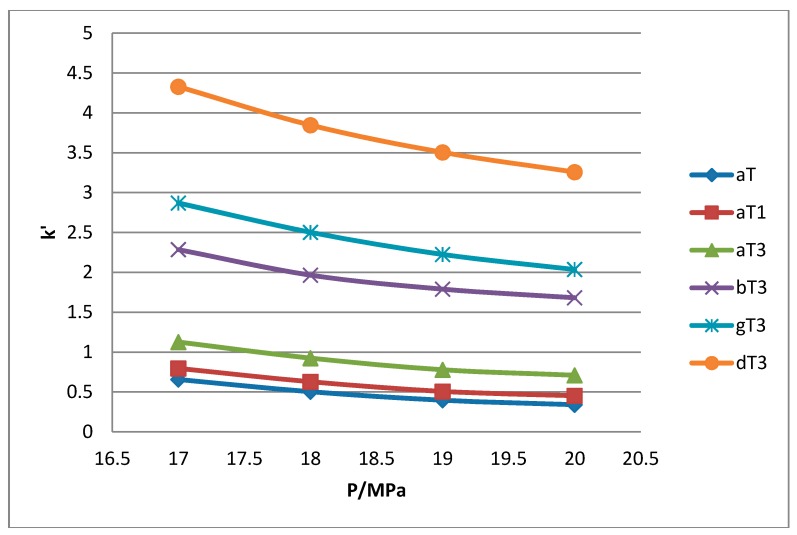
Effect of pressure on capacity factor for tocols dissolved in chloroform.

All the tocol analogs in palm oil are well resolved in SFC, however, baseline resolution between the α-T/α-T_1_ peaks was lost when the pressure was increased. Increase in pressure also resulted in a shorter elution time. Capacity factor, k’, decreased with increasing pressure as depicted in Figure 7, Figure 8, Figure 9 and Figure 10.

Table 1 shows the resolution of the αT/αT_1_ prepared in different injecting solvents, at different pressure while Table 2 shows the total elution time for the tocol analogs at different pressure.

## 3. Discussion

All the tocol analogs present in palm oil are eluted by supercritical CO_2_ modified with ethanol. The elution order of the tocol analogs was that the saturated analog eluted first, followed by the analogs in the order of increasing unsaturation. In normal phase chromatography, the polarity of the mobile phase and solute are the factors which determine the elution order. In this study, supercritical CO_2_, modified with a small percentage of an entrainer helped to elute the tocol analogs of moderate polarity.

Generally, samples for chromatographic analyses are dissolved in the same solvent as the mobile phase. In SFC, the mobile phase is a supercritical fluid. As such, samples are dissolved in other pre-selected solvents or the same solvent as a modifier. In this study, the mobile phase was modified with ethanol. Injecting solvents of moderate polarity or eluent strength such as hexane, heptane, dichloromethane and chloroform did not have a significant effect on the peak shapes of any of the tocol analogs. However, peak broadening was observed when ethanol was used as an injecting solvent. This was somewhat unexpected as the mobile phase itself contains ethanol. On the other hand, introducing a third component into the mobile phase in the form of injecting solvent did not have any effect on the SFC separation.

Solvents of eluent strength up to 3.4 are suitable for the use as injecting solvents when preparing the tocol analogs sample for SFC. Although in minimal amount, the high eluent strength of the injecting solvent does play a role in determining the peak shape and separation of the tocol analogs in palm oil.

The retention of the tocol analogs in SFC is described by the capacity factor, k’. k’ is expressed as k’ = (t_r_ − t_0_)/t_0_ where t_r_ is the retention time of the tocol while t_0_ is the dead time of the column, measured as the elution time of the injecting solvent. The injecting solvent was hardly retained on the column and thus, its retention time is used as the dead time (t_0_) in the calculation of the capacity factor. By keeping the temperature constant, the effect of pressure on the separation of the analogs can be clearly seen. It was observed that the capacity factors decreased with increasing pressure. The mobile phase is more compressible at a low pressure, resulting in a high capacity factor. The density of the mobile phase increases with pressure, thus enhancing the solubility of the solute in the mobile phase which, in turn, results in lower retention.

While the rest of the tocols are well separated, the αT/αT_1_ pair was not very well resolved and this is of particular interest where the effect of pressure is a concern. Generally, the resolution decreases with the increase in pressure. However, the pair is still well resolved up to 20 MPa. The total eluting time decreases with the increase of pressure as shown in Table 2. The density of mobile phase increases with pressure, resulting in a higher solubility of the tocol analogs in supercritical CO_2_. This in turn, facilitated the elution of the tocol analogs from the stationary phase, hence lower resolution and shorter elution time are observed.

## 4. Materials and Methods

### 4.1. Materials

All solvents used were of chromatographic grade, purchased from Merck (Darmstadt, Germany). Tocols were purchased from Carotino (M). Sdn. Bhd. (Johor, Malaysia). Carbon dioxide was of chromatographic grade (99.995%) and obtained from Malaysian Oxygen (Selangor, Malaysia).

SFC was carried out using a JASCO SFC system (JASCO, Easton, MD, USA) coupled with a photodiode array detector. The column used was silica (5 µm, 4.6 × 250 mm).

### 4.2. SFC of Tocols

Tocols (0.04 g) were weighed and dissolved in *n*-heptane, *n*-hexane, dichloromethane (DCM), ethanol or chloroform (5 mL), respectively. An aliquot of this mixture (20 µL) was then injected into the SFC instrument. The CO_2_ flowrate was 2.0 mL min^−1^ with 0.02 mL min^−1^ absolute ethanol as a modifier. The column temperature was set at 50 °C and pressure 17, 18, 19 and 20 MPa respectively. The procedure was repeated for pressure at 18, 19 and 20 MPa. Detection of the tocols was carried out at 290 nm.

## Figures and Tables

**Table 1:** Resolution between alpha-T and alpha-T_1_ at different pressure in samples dissolved in various injecting solvents.

Pressure (MPa)	Injecting Solvent				
	Heptane	Hexane	DCM	EtOH	Chloroform
17	1.106	1.099	0.978	0.907	0.973
18	1.058	0.827	0.949	1.249	0.978
19	0.955	0.854	0.864	0.916	0.910
20	0.942	0.870	1.000	0.726	0.758

**Table 2:** Total elution time for tocols at different pressure in samples dissolved in various injecting solvents.

Pressure (MPa)	Injecting Solvent				
	Heptane	Hexane	DCM	EtOH	Chloroform
17	55	55	55	55	55
18	51	52	50	48	50
19	47	47	47	47	47
20	46	44	46	44	44
